# Impact of organic pollutants on phenotype and gene expression in human breast cancer cells

**DOI:** 10.1002/jat.4961

**Published:** 2025-10-21

**Authors:** Camila Confortin, Heloisa Amancio, Jessica Zablocki da Luz, Aliciane de Almeida Roque, Tugstênio Lima de Souza, José Eduardo Vargas, Ciro Alberto de Oliveira Ribeiro, Francisco Filipak Neto

**Affiliations:** ^1^ Department of Cell Biology Federal University of Paraná (UFPR) Curitiba Brazil

**Keywords:** cancer, chronic exposure, in vitro study, organic pollutant, prognosis

## Abstract

Human exposure to industrial chemical compounds is widespread and, although often beneficial, prolonged contact may contribute to disease development, including cancer. While many studies have shown organic pollutants (OP) are cytotoxic, few have explored how long‐term exposure alters cell phenotype. Therefore, the current study investigated whether exposure to the OP perfluorooctanoic acid (Pfoa: 0.01, 0.1, and 1 μM), bisphenol A (Bpa: 0.1 and 1 μM), methoxychlor (Mtx: 0.1 and 1 μM), and benzophenone‐1 (Bp1: 1 and 10 μM) modulates the phenotype of MCF7 human breast cancer cells and MCF10A normal breast epithelial cells. MCF7 cells were exposed to environmentally relevant concentrations of the OP for 24 h and 15 days. Cell viability, proliferation, colony formation, drug‐efflux activity, and gene expression were assessed. A modulation of MCF7 breast cancer cell phenotype was observed, with increased proliferation and colony formation (Pfoa, Bpa, Mtx, Bp‐1), particularly at 15‐day exposure and with expression of genes involved in cell survival, proliferation, differentiation, and chemoresistance (STAT3 and VEGFA [Bpa, Mtx, Bp‐1], BRCA1 [Bp1], ESR2 [Pfoa, Bpa], ABCG2 [Pfoa]). No effects on cell migration and drug‐efflux activity were observed. Likewise, an increase in cell proliferation also occurred for MCF10A nontumor cells (Bpa, Mtx, Bp‐1), but these effects were usually less pronounced than those observed in MCF7 cells. Pan‐cancer analysis revealed a negative correlation between the expression of STAT3, VEGFA, ESR2, and ABCG2 and breast cancer patient survival. These findings suggest that low‐concentration, prolonged exposure to OP may promote tumor progression and aggressiveness in breast cancer, potentially undermining therapeutic outcomes.

## Abstract 2

This study assessed whether long‐term exposure to organic pollutants (PFOA, BPA, MTX, and BP‐1) modulates MCF7 breast cancer and MCF10A normal breast cell phenotype. At environmentally relevant concentrations, these compounds increased MCF7 cell viability, proliferation, colony formation, and expression of genes linked to survival, proliferation, and chemoresistance, especially after 15 days. MCF10A cells also showed increased proliferation, though less markedly. These findings suggest that prolonged exposure to such pollutants may influence tumor progression and potentially affect breast cancer outcomes.

## 1 Introduction

Breast cancer remains a leading cause of death in women, despite advances in diagnosis and treatment. It is biologically heterogeneous, influences clinical behavior, and response to several treatments. During the development of the disease, there is an uncontrolled proliferation of abnormal cells, forming a tumor, and later cell spreading through blood and lymphatic vessels. In this process, intratumoral heterogeneity, driven by gene expression reprogramming, plays a key role in cell survival and responsiveness to microenvironmental signals (Zheng et al. 2022; Clusan et al. 2023; Khan et al. 2022). Luminal breast epithelial cells, which express high levels of hormone receptors such as estrogen (ER) and progesterone receptors (PR), give rise to most breast cancers and are therefore particularly susceptible to endocrine disrupting chemicals (Clusan et al. 2023).

Environmental factors, including lifelong chemical exposure, may increase cancer risk (Oliveira et al. 2022) and alter tumor cell phenotype both in vitro (Silva Filho et al. 2022) and in vivo (Marchi et al. 2024). Many contaminants and pollutants to which humans are exposed act as endocrine disruptors, interacting with hormone receptors and mimicking their effects. For instance, bisphenol A (Bpa) interacts with estrogen receptors alpha and beta (Sweeney et al. 2015; Wang et al. 2025); perfluorooctanoic acid (Pfoa) mimics fatty acids, disrupting hormonal balance (Basini et al. 2022; Santaliz Casiano et al. 2022); phenolic compounds such as benzophenones affect the endocrine system (Xiong et al. 2025); and lipophilic organochlorines like methoxychlor (Mtx) alter hormonal homeostasis (Miranda et al. 2022; Kumar et al. 2023).

Studies assessing environmentally relevant concentrations of pollutants are crucial for evaluating human health risks, including cancer (Hammel et al. 2024; Rawn et al. 2024; Bogdan et al. 2024). In this regard, most studies have focused on the carcinogenicity of chemicals (Huang et al. 2015; Lecomte et al. 2017; Sweeney et al. 2015; Koual et al. 2020), whereas their effects on tumor cell phenotype and progression remain underexplored (De Almeida Roque et al. 2023; Steil et al. 2022; Silva Filho et al. 2022; Montalbano et al. 2020; Xue et al. 2022; De Marchi et al. 2021; Marchi et al. 2024; Qi et al. 2024). This approach is relevant because pollutant exposure can modulate gene expression, increasing tumor aggressiveness and chemoresistance (De Almeida Roque et al. 2023).

The current study, therefore, aimed to determine whether a group of organic pollutants modulates the phenotype of MCF7 breast cancer cells. MCF7, a luminal A ER+/PR+ cell line, is a good model of primary breast tumors, whereas MCF10A serves as a nontumor reference. The terms (contaminant, pollutant, breast cancer, cell proliferation, etc.) were used as keywords for the literature search. The compounds tested were perfluorooctanoic acid (PFOA), used in waterproof clothing and nonstick cookware (Zhang et al. 2014); bisphenol‐A (BPA), found in food and beverage cans and bottles (Daronch et al. 2020); methoxychlor (MTX), an insecticide used in home gardens and for pets (Chen 2014); and benzophenone‐1 (BP1), used in sunscreen and cosmetics (Zou et al. 2021). These chemicals will be referred to hereafter as organic pollutants (OP). Concentrations were selected based on levels typically found in human tissues and fluids.

Cells were exposed for 24 h and 15 days. Unlike most studies that use high concentrations for short periods, we employed prolonged exposure to concentrations closer to real‐life human exposure. The 24‐h period identifies immediate cellular responses, whereas the 15‐day period models chronic exposure, allowing the assessment of early phenotypic changes such as treatment resistance, proliferation, migration, invasion, and gene expression alterations associated with tumor behavior. This design provides a comprehensive understanding of the effects of OP on breast tumor cells.

## 2 Material and Methods

### 2.1 Selection of the Organic Pollutants (OP) and Molecular Docking

The OP was selected based on data found in literature and molecular docking. First, we searched for papers in the literature reporting effects of chemical contaminants and pollutants in breast, ovarian, and prostate cancer cells, particularly in processes important for cancer, such as cell proliferation, migration, invasion, drug‐efflux, epithelial‐mesenchymal transition, and expression of key molecules, for example, matrix metalloproteinases and their inhibitors, cell cycle proteins, and so on. We initially selected 23 chemicals with effects in cancer cells, but we then reduced the number of compounds to the 10 most promising ones, based on the relevance (biological significance, robustness, and importance of the effects for cancer) of the data. In particular, the studies of Kim et al. 2014, 2015; Lee et al. 2012; Park et al. 2013; Li et al. 2018; Zhang et al. 2014, 2019 reported important data for the selection of the chemicals.

Then, we performed molecular docking calculations between these 10 chemicals (triclosan, octylphenol, Mtx, Bp1, endosulfan, Bpa, lindane, hexachlorobenzene, Pfoa, and parathion) and the crystallographic model of 32 proteins obtained from the Protein Data Bank (PDB) using the AutoDock Vina program (Trott and Olson 2010). These proteins were selected based on their role in tumor progression and in processes important for cancer: AKT (protein kinase B), cyclin D1, Bcl‐2 (B‐cell lymphoma 2), BclXL (B‐cell lymphoma extra‐large), MMP2 (matrix metalloproteinase 2), MMP9 (matrix metalloproteinase 9), survivin, vimentin, N‐cadherin, ERK2 (extracellular signal‐regulated kinase 2), ESR1 (estrogen receptor 1), EGFR (epidermal growth factor receptor), CDK2 (cyclin‐dependent kinase 2), Ras 1 (Ras proto‐oncogene 1), c‐RAF (a serine/threonine kinase), P38 (p38 mitogen‐activated protein kinase), Mdm2 (mouse double minute 2 homolog), mTOR (mechanistic target of rapamycin), VEGF (vascular endothelial growth factor), TGFB1 (transforming growth factor beta 1), MDR1 (multidrug resistance protein 1), NF‐κB (nuclear factor kappa B), CDC25 (cell division cycle 25), CDC42 (cell division cycle 42), PI3K (phosphoinositide 3‐kinase), ATM (ataxia telangiectasia mutated), RhoA (Ras homolog family member A), ROCK (rho‐associated coiled‐coil containing protein kinase), PR (progesterone receptor), HER2 (human epidermal growth factor receptor 2), BCRP (breast cancer resistance protein), and GPR30 (G protein‐coupled receptor 30).

The selection of the compounds for the in vitro experiments was performed via molecular screening with parameters defined using AutoDock tools (Morris et al. 2009). The affinities were selected based on Montes‐Grajales et al. (2020), using the cut‐off value of −7.0 kcal/mol, representing approximately 95% of the specificity. Based on the best binding scores, that is, values greater than −7.0 kcal/mol (high affinity binding), four compounds were selected for this study (Pfoa, Bpa, Mtx, and Bp1).

Pfoa is not metabolized by the body, has a human half‐life of 4–5 years, and has been detected in blood (7–9370 ng/mL; Pierozan et al. 2018) and serum (~4 ng/mL; Wang et al. 2024). Bpa has been found in urine (2.5–4.5 μg/dm^3^), blood (1–4 μg/dm^3^), breast milk (1.1–3.4 μg/dm^3^), placenta (1–104 μg/kg), and adipose tissue (3.16 μg/kg) (Michałowicz 2014). Mtx is an insecticide linked to endocrine disruption (Bhattacharya and Keating 2012), and has been detected in placenta, adipose tissue, breast milk, and serum (Botella et al. 2004; Shen et al. 2007) and can persist in the environment (Witek et al. 2021). Bp‐1 has been detected in urine, blood, amniotic fluid, umbilical cord, and placenta (Zhang et al. 2013; Kang et al. 2016; Krause et al. 2018; Lu et al. 2018; Mao et al. 2022).

### 2.2 Acquisition of Contaminants

These chemicals were purchased from Sigma‐Aldrich (95%–99% pure, Pfoa: 171468‐5G, Bpa: 239658‐50G, Mtx: 36161‐100MG, Bp1: 126217‐100G), and then stock solutions were prepared in pure DMSO (dimethylsulfoxide), diluted in PBS (phosphate buffered saline; 2000× concentrated), and stored in amber glasses at −20°C. From these stocks, 500× concentrated solutions were made in DMSO and PBS, and then diluted in supplemented culture medium used to expose the cell (final DMSO concentration in culture medium = 0.05%).

### 2.3 Cell Culture

The culture medium for MCF7 and MCF10A cells was prepared following the guidelines of the *Banco de Células do Rio de Janeiro* (BCRJ 2021). MCF7 cells were cultured in RPMI 1640 (Gibco) culture medium supplemented with 10% fetal bovine serum (FBS), 0.01 mg mL^−1^ human insulin, and 40 mg mL^−1^ gentamicin. MCF10A cells were cultured in DMEM/F12 (Gibco) medium (1:1) with 2.5 mM L‐glutamine, 5% horse serum, 10 μg mL^−1^ human insulin, 0.5 μg mL^−1^ hydrocortisone, 10 ng mL^−1^ EGF (epidermal growth factor), 100 ng mL^−1^ cholera toxin, and 40 mg mL^−1^ gentamicin. Both cell lines were kept at 37°C in an incubator with a humidified atmosphere and 5% CO_2_. Subcultures were performed every 4 days after cells were trypsinized (0.25% trypsin, 0.02% ethylenediaminetetraacetic acid [EDTA]), centrifugation (123 g), and seeding.

### 2.4 Experimental Design

The cells were exposed to the organic pollutants (Pfoa: 0.01, 0.1, and 1 μM; Bpa: 0.1 and 1 μM; Mtx: 0.1 and 1 μM; and Bp‐1: 1 and 10 μM) in the culture medium (supplemented as described in the Cell Culture section) for 24 h (24 h experiment) and 15 days (15d experiment). For each experiment, a control group was included. This group received the supplemented culture medium with DMSO, the vehicle used for contaminant exposure. For the 24 h experiment, cells were seeded on culture plates, cultured for 24 h to ensure attachment and recovery, exposed to the organic pollutants by medium replacement for 24 h, and then used for the assays. For the 15d experiment, 8 × 10^4^ cells were seeded in 25 cm^2^ culture flasks, cultured for 24 h, and then exposed to the pollutants. On days 4, 8, and 12 after seeding, cells were trypsinized and transferred to new culture flasks with fresh medium containing the pollutants to maintain continuous exposure throughout the 15‐day period. On day 15 after seeding (i.e., after 14 days of continuous exposure to OP), cells were trypsinized and seeded into appropriate culture vessels (9‐, 24‐, and 96‐well plates or 25 cm^2^ flasks), depending on the assay. They were cultured for 24 h in OP‐free medium to allow attachment and recovery from trypsinization, a step intended to standardize conditions and reduce variability before the final assays. The cells were then re‐exposed to the same pollutant concentrations for 24 h, completing the 15‐day exposure protocol. For both experiments, a control group underwent the same procedures without pollutant exposure.

### 2.5 Cell Viability

Cell viability and cytotoxicity were assayed in 96‐well microplates (1.5 × 10^4^ cells seeded per well) by neutral red, MTT, and crystal violet assay at 24 h and 15 d experiments. The absorbance of the cell viability experiments was measured using the Varioskan Lux equipment from Thermo Scientific.

### 2.6 Neutral Red

The cells were incubated with 50 μg mL^−1^ in culture medium at 37°C for 2 h, washed twice with PBS, and fixed with 100 μL of 4% formaldehyde. The dye was extracted with 300 μL of 49.5% H_2_Od, 49.5% ethanol, and 1% glacial acetic acid, and 200 μL was transferred to a new plate for absorbance measurement at 540 nm (Ates et al. 2017).

### 2.7 MTT

The cells were incubated with 0.5 mg mL^−1^ of MTT (3‐(4,5‐dimethylthiazol‐2‐yl)‐2,5‐diphenyltetrazolium bromide) at 37°C for 1 h, and washed twice with PBS. The formazan crystals were solubilized with 100 μL of DMSO and the absorbance measured at 550 nm (Grela et al. 2018).

### 2.8 Crystal Violet

The cells were washed with PBS, fixed with 100 μL of methanol for 10 min, and stained with 50 μL of crystal violet (0.25 mg mL^−1^) for 10 min at room temperature. The wells were washed twice with PBS, the dye was extracted with 33% acetic acid under agitation for 30 min, and the absorbance was measured at 570 nm (Feoktistova et al. 2016).

### 2.9 Drug Efflux Transporters Activity

The activities of the drug‐efflux pumps PgP (P‐glycoprotein), MRPs (multidrug resistance‐associated proteins), and BCRP (breast cancer resistance protein) were assayed in cells cultured in 96‐well microplates (1.5 × 10^4^ cells seeded per well) and incubated with fluorescent solutes at 24‐h and 15‐d experiments. The absorbance of the experiments assessing the activity of drug efflux transporters was measured using the Varioskan Lux equipment from Thermo Scientific.

After the exposure period, the cells were incubated with 100–200 μL of medium containing one of the fluorescent solutes (1 μM rhodamine B for PgP/MRPs, 0.5 μM calcein‐AM for MRPs, and 0.5 μM hoechst 33342 for BCRPs). Following incubation for 0.5–1 h, the wells were washed twice with PBS and incubated with 200 μL of PBS for 10 min at 37°C. The supernatants were transferred to a black microplate, and the fluorescence intensity was measured (rhodamine B: λex/em = 540/580 nm, calcein‐AM: λex/em = 488/530 nm, hoechst 33342: λex/em = 460/490 nm). All the procedures described, starting with incubation with the fluorescent solutes, were performed protected from direct light. Cells concomitantly incubated with the fluorescent solutes and transporter inhibitors were used as positive controls (PgP/MRPs: 60 μM verapamil, MRPs: 25 μM indomethacin, BCRPs: 25 μM elacridar) (Liebel et al. 2015, De Souza Salgado et al. 2018, Seigel and Campbell 2004, Hooijberg et al. 2004; Sato et al. 2015; De Marchi et al. 2021).

### 2.10 Cell Migration

Wound healing/scratch assay was carried out according to Liang et al. (2007) with minor modifications in 96‐well microplates (5 × 10^4^ cells seeded per well) at the 15d experiment. The culture medium was replaced with a fresh SFB‐free medium containing mitomycin C (10 μg mL^−1^). After 2 h incubation, a scratch was made in the center of each well with a 10 μL micropipette tip, the wells were washed twice and filled with FBS‐free culture medium. Images were captured on an inverted microscope (Leica Microsystems) after 0, 24, and 48 h. The closure of the scratch was determined using ImageJ software (http://imagej.nih.gov/ij/).

### 2.11 Clonogenic Assay

Survivability at low density and colony formation on solid substrate were assessed as described by Franken et al. (2006), with some modifications. The cells were plated in 24‐well plates (150 cells seeded per well) in 24 h‐ and 15 d‐experiments. After exposure, the culture medium was replaced with fresh culture medium without the OP, and the cells were cultured at 37°C for 6 days, with medium replacement every 3 days. Then, the colonies were fixed with 4% paraformaldehyde in PBS for 30 min, washed with PBS, stained with crystal violet (0.25 mg mL^−1^) for 15 min, and washed again with PBS. Finally, images of the colonies were captured using a Discovery.V12 stereomicroscope (Zeiss) for colony counting (clusters of 50 or more cells) using ImageJ software.

### 2.12 Cell Proliferation

Cell proliferation was analyzed using CellTrace Far Red‐APC (ThermoFisher Scientific) following the manufacturer's instructions at the 15d‐experiment. For the assay, 10^5^ cells were suspended and washed with PBS in 15 mL centrifuge tubes, and incubated with CellTrace for 20 min at 37°C and protected from light. The volume was then topped up with 5 mL of culture medium, the cells were incubated for 5 min, centrifuged (189 g for 3 min), resuspended in culture medium, and seeded and cultured in 6‐well plates at 37°C and 5% CO_2_ for 5 days. Then, the cells were trypsinized, fixed with 1 mL of 4% paraformaldehyde in PBS for 30 min, centrifuged, and resuspended in 1 mL of PBS for analysis by flow cytometry using red excitation at 630 nm and emission at 661 nm. FCS Express Research and FlowJo 7.6.2 software were used for analysis. The cell proliferation index is automatically calculated by various proliferation fitting statistics based on the data entered into the FCS Express software. It represents the average number of cells that a single starting cell has become. It is calculated using the following equation:

$$\sum_{i=0}^{P-1}Nᵢ÷\sum_{i=0}^{P-1}\frac{Nᵢ}{2^{i}}$$

where

***P*** is the total number of peaks detected (where **P₀** is the undivided generation);

***N*** is the number of cells in a given generation;

***μ*** is the average of a population.

### 2.13 Gene Expression

A total of 8 × 10^6^ cells were collected from the 15d‐experiment, resuspended in RNAlater (Invitrogen, ref. AM7021), and stored at −80°C. For RNA extraction, the cells were centrifuged at 189 g for 3 min at room temperature, and the total RNA was extracted using Trizol and chloroform, followed by a second step of purification using the PureLink RNA mini Kit (Ref. 12183018A‐Invitrogen). The RNA was quantified by fluorimetry (λex/em = 492/540 nm) using the QuantiFluor RNA System (Ref. E3310 [Promega]), and the purity was assessed by spectrophotometry (λ = 230, 260, and 280 nm) for protein contamination (260/280 nm ratio) and phenol and other contaminants (260/230 nm ratio), with limit values of ∼2.0 and 1.8–2.2, respectively (Desjardins and Conklin 2011). RNA integrity was verified by electrophoresis in a 1% agarose gel. The conversion of RNA into cDNA was carried out using the SuperScript IV First‐Strand Synthesis System (Invitrogen, ref. 18,091,050) and Veriti Thermal Cycler. For RT‐qPCR, the PowerUp SYBR Green Master Mix (Ref. A25776‐Applied Biosystems) and the StepOnePlus Real‐Time PCR System (Ref. 4.376.600‐Applied Biosystems) were used, set up for recommended thermocycling conditions. For each 10 μL reaction, 10 ng of cDNA and 500 nM of primers (Table 1) were used. The absence of DNA contamination from other sources or the presence of primer dimers was verified through the no template control (NTC) strategy, in which the template DNA was omitted from the reactions. The data was processed using the LinRegPCR server (https://www.gear‐genomics.com/rdml‐tools/index.html, Untergasser et al. 2021). The expression of a group of genes (Table 1) that are associated with important processes in breast tumorigenesis was investigated.

**TABLE 1:** Primers used in RT‐qPCR.

Gene	Primers sequences (5′‐3′)	Amplicon	RefSeq accession
HSPA8	F: 5′‐ACCTACTCTTGTGTGGGTGTT‐3′ R: 5′‐GACATAGCTTGGAGTGGTTCG‐3′	87pb	NM_006597.6
ABCG2	F: 5′‐TGGCTGTCATGGCTTCAGTA‐3′ R: 5′‐GCCACGTGATTCTTCCACAA‐3′	206pb	NM_004827.3
AKT1	F: 5′‐GTCATCGAACGCACCTTCCAT‐3′ R: 5′‐AGCTTCAGGTACTCAAACTCGT‐3′	218pb	NM_005163.2
BRCA1	F: 5′‐CTGAAGACTGCTCAGGGCTATC‐3′ R: 5′‐AGGGTAGCTGTTAGAAGGCTGG‐3′	152pb	NM_001408467.1
STAT3	F: 5′‐TCCATCAGCTCTACAGTGACAGC‐3′ R: 5′‐TCCCAGGAGATTATGAAACACC‐3′	134pb	NM_001384993.1
VEGFA	F: 5′‐CTACCTCCACCATGCCAAGT‐3′ R: 5′‐GCAGTAGCTGCGCTGATAGA‐3′	109pb	NM_001025366.3
β‐TUBULIN	F: 5′‐CACAGGCAGTTACCATGGAG‐3′ R: 5′‐GTCTGAAGATCTGGCCGAAG‐3′	164pb	NM_001310315.2
MMP2	F: 5′‐AGCGAGTGGATGCCGCCTTTAA‐3′ R: 5′‐CATTCCAGGCATCTGCGATGAG‐3′	138pb	NM_001302510.2
MMP9	F: 5′‐GCCACTACTGTGCCTTTGAGTC‐3′ R: 5′‐CCCTCAGAGAATGCCAGTACT‐3′	124pb	NM_004994.3
ANKRD17	F: 5′‐AATGTTGCCACCACTCTTCC‐3′ R: 5′‐TGCAGCTGTGCATTCTTTTC‐3′	200pb	NM_001286771.3
GAPDH	F: 5′‐ACAACTTTGGTATCGTGGAAGGAC‐3′ R: 5′‐CAGGGATGATGTTCTGGAGAGC‐3′	129pb	NM_001357943.2
B‐ACTIN	F: 5′‐CACCATTGGCAATGAGCGGTTC‐3′ R: 5′‐AGGTCTTTGCGGATGTCCACGT‐3′	135pb	NM_001101.5
AR	F: 5′‐CCCATCTATTTCCACACCCA‐3′ R: 5′‐GCAAAGTCTGAAGGTGCCAT‐3′	259pb	NM_000044.3
RPS6	F: 5′‐CATGAAGCAGGGTGTCTTGA‐3′ R: 5′‐ACAATGCAACCACGAACTGA‐3′	120pb	NM_001010.3
SYNCRIP	F: 5′‐CTGGTCTCAATAGAGGTTATGCG‐3′ R: 5′‐TCCGGTTGGTGGTATAAAATGAC‐3′	244pb	NM_001410938.1
ESR1	F: 5′‐ACTGCGGGCTCTACTTCATC‐3′ R: 5‐GGCTGTTCCCAACAGAAGAC‐3′	275pb	NM_000125.3
ESR2	F: 5′‐CTCTTTTGCCTGAAGCAACG‐3′ R: 5′‐CTGGGCAGTTAAGGAGACCA‐3′	269pb	NM_001040275.1

*Note:* Primers: HSPA8 (Annamaneni et al. 2019), ABCG2 (Jing et al. 2020), AKT1 (Mohamadzade et al. 2021), BRCA1 (Chatterjee et al. 2019), STAT3 (Zhang et al. 2011), VEGFA (Kalinina et al. 2021), β‐TUBULIN (Saussede‐Aim et al. 2009), MMP2 (Wang et al. 2021), MMP9 (Wang et al. 2021), ANKRD17 (Kalinina et al. 2021), GAPDH (Kalinina et al. 2021), β‐ACTIN (Wang et al. 2021), AR (Stecca et al. 2016), RPS6 (Hu et al. 2017), SYNCRIP (Zhang et al. 2020), ESR1 (Stecca et al. 2016), ESR2 (Stecca et al. 2016), and SYMPK (Tilli et al. 2016).

### 2.14 Tumor Data Analysis

A pan‐cancer analysis was performed using approximately 9000 transcriptomic profiles from The Cancer Genome Atlas (TCGA) and the Cancer Cell Line Encyclopedia (CCLE) to assess the suitability of cell lines as models of primary tumors. Methodological details of the data processing were previously published by Yu et al. (2019). For breast cancer, a total of 1042 samples were analyzed, and Spearman correlation was performed between breast cancer cell lines and human breast cancer patients. This data is available on the UCSF website (https://comphealth.ucsf.edu/app/tcga‐110).

Gene expression and Kaplan–Meier survival analysis of BRCA1, ESR2, STAT3, and VEGF genes were performed using data from breast cancer patients. The UALCAN web resource (http://ualcan.path.uab.edu) was used for this analysis.

### 2.15 Statistical Analysis

Five independent experiments were conducted for each in vitro assay, using 5–6 replicates per experiment and group. The mean values of the replicates of each independent experiment were used to compare the OP‐exposed groups and control. The assumptions of normality and homoscedasticity were tested using the Shapiro–Wilk and Brown–Forsythe tests, respectively. Parametric data were analyzed using one‐way ANOVA, followed by Dunnett's test and presented as means. Nonparametric data were evaluated using the Kruskal–Wallis test, followed by Dunn's multiple comparisons test, and presented as medians. A *p* < 0.05 was considered statistically significant.

## 3 Results

### 3.1 Selection of the Organic Pollutants (OP)

The selection of chemical compounds for in vitro testing was performed via molecular screening with parameters defined using AutoDock tools. Molecular docking was performed between 10 pre‐selected compounds and 32 protein targets important for tumor phenotype and progression. We selected the 4 compounds with the largest number of high and moderate affinity protein targets (Table 2), that is, binding energies above −7.0 kcal/mol (high affinity), and between −6.9 and −6.0 kcal/mol (moderate affinity, Montes‐Grajales et al. 2020). Pfoa was the compound with the largest number of high (20) and moderate (10) affinity targets, followed by Bpa (high/moderate affinity: 11/16 targets), Bp1 (high/moderate affinity: 7/19 targets), and Mtx (high/moderate affinity: 6/15 targets, Table 2).

**TABLE 2:** Molecular docking of the 10 pre‐selected compounds and protein targets.

Targets	Id. PDB	Triclosan	Octylphenol	Mtx	Bp1	Endosulfan	Bpa	Lindane	Hexachlorobenzene	Pfoa	Parathion
1‐AKT	1Q0R	−5.1	−5.7	−5.8	−6.5	−6	−6.8	−4.6	−6.2	−7.1	−5.5
2‐Cyclin D1	3AY5	−5.2	−5.5	−6.2	−6.3	−5.2	−7.2	−4.1	−4.2	−7.7	−5
3‐Bcl‐2	6VO4	−5.3	−5.2	−6.5	−6.5	−6	−6.5	−4.6	−4.3	−6.8	−4.8
4‐BclXL	7Y8D	−5.2	−5.7	−6.7	−6.5	−5.3	−7.3	−4.4	−4.6	−6.9	−5.8
5‐MMP2	1RTG	−5.4	−6.4	−7.2	−6.9	−6.8	−7.1	−4.9	−5.3	−7.1	−5.7
6‐MMP9	1L6J	−5.9	−6	−5.9	−6.4	−5.8	−6.4	−5.5	−5.8	−7.5	−5.7
7‐Survivin	1F3H	−5.6	−6.6	−5.6	−5.4	−6	−5.6	−4.5	−4.6	−6.6	−6
8‐Vimentin	4YPC	−4.6	−6.3	−6.4	−6.9	−7	−7	−5.4	−5.2	−8.1	−5.6
9‐N‐cadherin	4ZT1	−5.9	−4.9	−6	−5.8	−5.6	−6.2	−4.1	−4.7	−6.6	−5.3
10‐ERK2	4G6N	−4.8	−5.6	−6.1	−6.1	−6.6	−6.6	−3.8	−4.4	−7.3	−5.5
11‐ESR1	2OUZ	−4.6	−5.2	−5.2	−7.4	−5	−5.5	−5.7	−5.7	−6.2	−5.3
12‐EGFR	2GS2	−3.6	−3.9	−5.4	−4.9	−4.9	−5.6	−3.7	−3.6	−5.6	−4.6
13‐CDK2	1 W98	−5.6	−5.2	−6.6	−6.9	−6.5	−6.4	−4.6	−5.3	−8.4	−5.7
14‐Ras 1	6CERA	−4.6	−5.2	−6.1	−6.7	−5.4	−7.6	−4.2	−4.1	−7.1	−5.1
15‐c‐RAF	3OMV	−4.9	−5.4	−5.7	−8	−6.2	−6.9	−4	−5.8	−7.9	−5.3
16‐P38	1WFC	−5	−5.3	−6.2	−6.1	−6.2	6.6	−4.8	−5.2	−7.1	−5.4
17‐Mdm2	4HFZ	−6.1	−6.1	−6.8	−7	−5.6	−7.7	−4.6	−4.4	−7.3	−5.6
18‐mTOR	3JBZ	−5.6	−5.8	−6.8	−6.1	−6.8	−6.5	−5	−5.6	−7.1	−6
19‐VEGF	1VR2	−4.1	−5	−4.7	−5.8	−5.2	−6	−3.9	−4.1	−6.1	−5.4
20‐TGFB1	5VQP	−5.3	−5.2	−6.5	−6.5	−5.3	−8.1	−5.4	−4.8	−7	−5.6
21‐MDR1	2CBZ	−4.7	−6.8	−7.5	−7.3	−5.6	−6.7	−4.7	−5.4	−7.1	−5.1
22‐NF‐κB	4OT9	−5	−5.9	−5.7	−6.2	−5.6	−6.2	−4.3	−5	−6.9	−6.2
23‐CDC25	1C25	−4.6	−4.2	−4.9	−5	−5.4	−6.2	−4.2	−4.4	−5.5	−5.7
24‐CDC42	1KZG	−5.2	−6.9	−7.8	−7.6	−5.9	−7.5	−5.1	−5.5	−8.4	−5.4
25‐PI3K	7PG5	−6.7	−6.2	−6.3	−6.7	−6.5	−5.6	−5.5	−5.6	−7.6	−5.7
26‐ATM	6K9K	−6.5	−6.6	−7.4	−7.4	−7.3	−7.6	−5.3	−5.4	−8.6	−6.3
27‐RhoA	1A2B	−4.9	−4.8	−5.4	−5.1	−5.1	−5.6	−4.1	4.2	6.4	−4.6
28‐ROCK	7JOU	−5.4	−6.1	−7.2	−6.8	−5.3	−6.4	−5.4	−5.3	−7.7	−5.3
29‐PR	1A28	−4.9	−6.7	−6.8	−6.7	−6.2	−7	−6.2	−4.4	−8.8	−5.6
30‐HER2	3PP0	−5.3	−7.3	−7.2	−6.1	−5.8	−7.6	−5.8	−5.9	−7.2	−5
31‐BCRP	5NJ3	−5.4	−5.4	−5.8	−8.4	−6.5	−6.6	−4.7	−6.3	−6.9	−6
32‐GPR30	Q63ZY2	−5.7	−5.7	−6.1	−6.2	−5.8	−6.4	−4.6	−4.8	−6.8	−5.3
**Reference values**		**−3.0 to −4.9**		**−5.0 to −5.9**		**−6.0 to 6.9**		**−7.0 to −8.9**		**kcal/mol**	

*Note:* Binding energy values of −7.0 kcal/mol or higher are considered high affinity (red color), values from −6.9 to −6.0 kcal/mol are considered moderate affinity (pink color), and values below −5.9 kcal/mol are considered low affinity and/or non‐binding (blue color). Screening performed with Autodock Vina.

Abbreviations: Bp1: benzophenone‐1; Bpa: benzophenone‐A; Mtx: methoxychlor; Pfoa: perfluorooctanoic acid.

This approach provided a more rational selection of the OP for in vitro testing, while also identifying some of the potential targets and mechanisms for further investigation.

### 3.2 Exposure to the OP Increased Cell Proliferation and Sensitivity in MCF10A Cells

**FIGURE 1:**
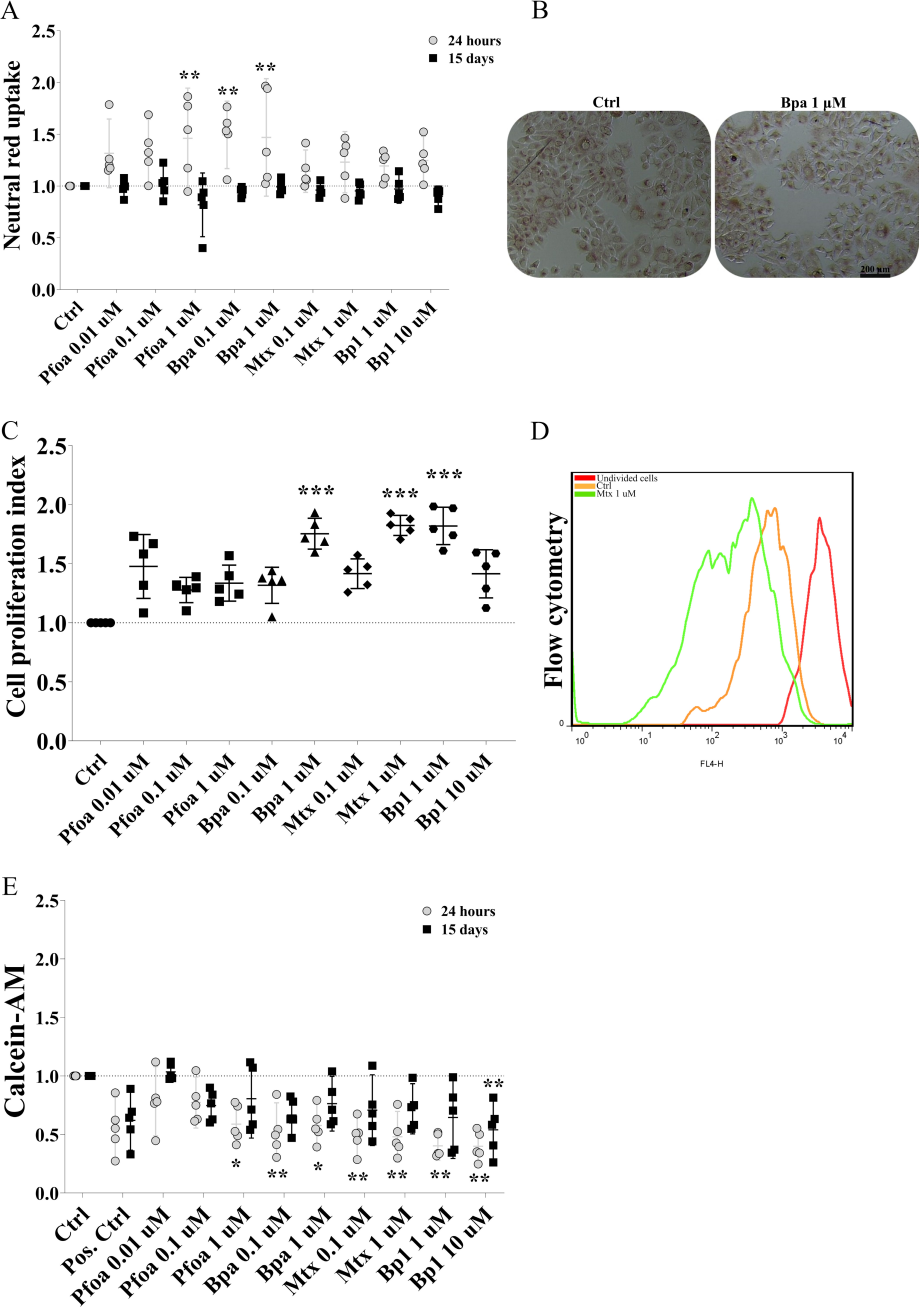
Cell viability, proliferation and drug efflux activity in MCF10A nontumor cells. (A) cell viability/neutral red (NR) uptake. (B) MCF10A cells with retained neutral red. (C) Cell proliferation index, chronic exposure. (D) Flow cytometry histogram of MCF10A cells labeled with CellTrace Far Red‐CFTR (red line: cells before any cell division, orange line: control (cells not exposed to the pollutants), green line: cells exposed to Mtx‐1 μM). (E) drug efflux assay with calcein AM to assess MRP activity. Gray circles: 24 h‐experiment. Black squares: 15d‐experiment. Horizontal line: mean or median, vertical line: SD (or 95% CI). One‐way ANOVA + Dunnett's test (A, E) or Kruskal‐Wallis + Dunn's (C) post hoc test to compare treated cells with the respective control (dashed horizontal line). *p < 0.05, **p < 0.01, ***p < 0.001. *N* = 5 independent experiments (circles and polygons). Scale bar = 200 μm in (B).

The neutral red (NR) uptake assay revealed ~40% increases in MCF10A nontumor cells exposed to Pfoa‐1 μM, Bpa‐0.1 μM, and Bp1‐1 μM at the 24 h experiment (Figure 1A,B), but no effects occurred for the crystal violet (CV) assay or decreases in the MTT metabolism assay (Figure S1). Increases in cell proliferation were found in cells exposed to Bpa‐1 μM (~70%), Mtx‐1 μM (~80%), and Bp1‐1 μM (~80%) at the 15 d experiment (Figure 1C,D).

Decreases in drug efflux pump activity using calcein‐AM, which is mainly related to the activity of the MRP proteins, were observed in cells exposed to Pfoa‐1 μM (~50%), Bpa‐0.1 μM and 1 μM (~50%), Mtx‐0.1 μM and 1 μM (~40%), and Bp1‐1 μM and 10 μM (~40%) at the 24 h experiment (Figure 1E). Likewise, a ~50% decrease in transporters' activity was observed in cells exposed to Bp1‐10 μM at the 15 d experiment (Figure 1E). However, transporters' activity using rhodamine B and Hoechst 33342 was not affected by exposure (Figure S1). Taken together, we observed that the OP had no cytotoxic effect on MCF10A nontumor cells and on cell migration (Figure S3), but decreased efflux pump activity, which may make these cells more sensitive to chemicals exported by these proteins, and increased cell proliferation.

### 3.3 Exposure to the OP Increased Cell Viability and Attachment in MCF7 Cells

**FIGURE 2:**
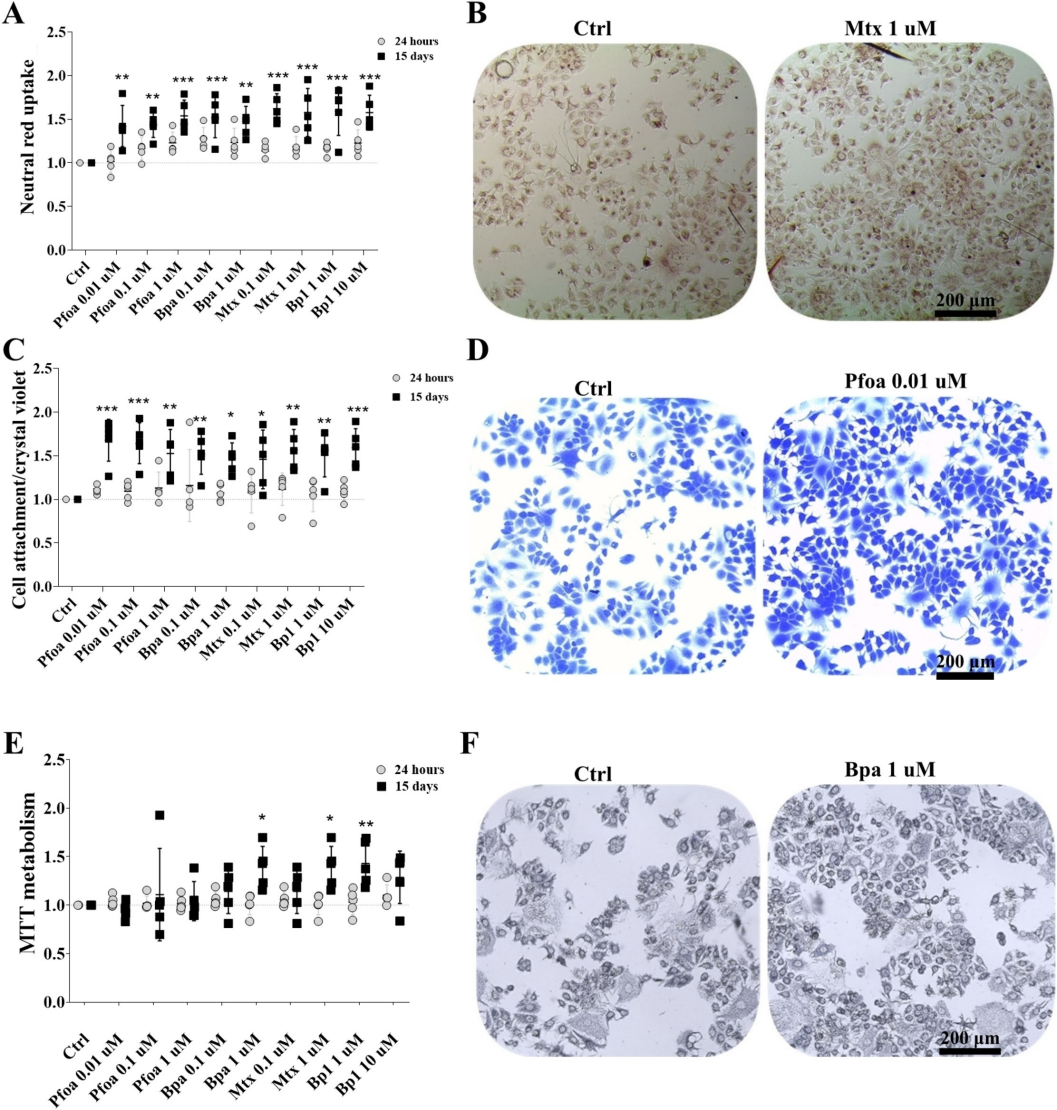
Cell viability and attachment in MCF7 tumor cells. (A) Neutral red uptake (NR). (B) MCF7 cells with retained neutral red. (C) Cell attachment/crystal violet (CV) assay. (D) MCF7 cells stained with crystal violet. (E) MTT metabolism (MTT). (F) MCF7 cells with formazan crystals. Gray circles: 24 h‐experiment. Black squares: 15d‐experiment. Horizontal line: mean, vertical line: SD (or 95% CI). One‐way ANOVA + Dunnett's test post hoc test to compare treated cells with the respective control (dashed horizontal line). *p < 0.05, **p < 0.01, ***p < 0.001. *N* = 5 independent experiments (circles and squares). Scale bar = 200 μm.

NR increased in MCF7 tumor cells exposed to Pfoa‐0.01 μM (~40%), 0.1 μM (~40%), and 1 μM (~50%), Bpa‐0.1 μM (~50%) and 1 μM (~40%), Mtx‐0.1 μM (~60%) and 1 μM (~50%), and BP1‐1 μM and 10 μM (~50%) at the 15d‐experiment (Figure 2A,B). CV revealed increases in the number of attached cells following exposure to Pfoa‐0.01 μM (~60%), 0.1 μM (~60%), and 1 μM (~50%), Bpa‐0.1 μM (~50%) and 1 μM (~40%), Mtx‐0.1 μM (~40%) and 1 μM (~50%), and BP1‐1 μM and 10 μM (~50%) at the 15d‐experiment (Figure 2C,D). Likewise, increases in MTT metabolism were found in cells exposed to Bpa‐1 μM (~30%), Mtx‐1 μM (~30%), and BP1‐10 μM (~40%) at the 15d‐experiment (Figure 2E,F). No changes were observed at the shorter exposure time (24 h‐experiment).

In short, considering NR, CV, and MTT assays altogether, it was observed that the OP had no cytotoxic effect on MCF7 tumor cells but led to increases in both the cell viability and the number of attached cells at long‐term exposure.

### 3.4 Exposure to the OP Increased Cell Proliferation and Expression of STAT3 in MCF7 Cells

**FIGURE 3:**
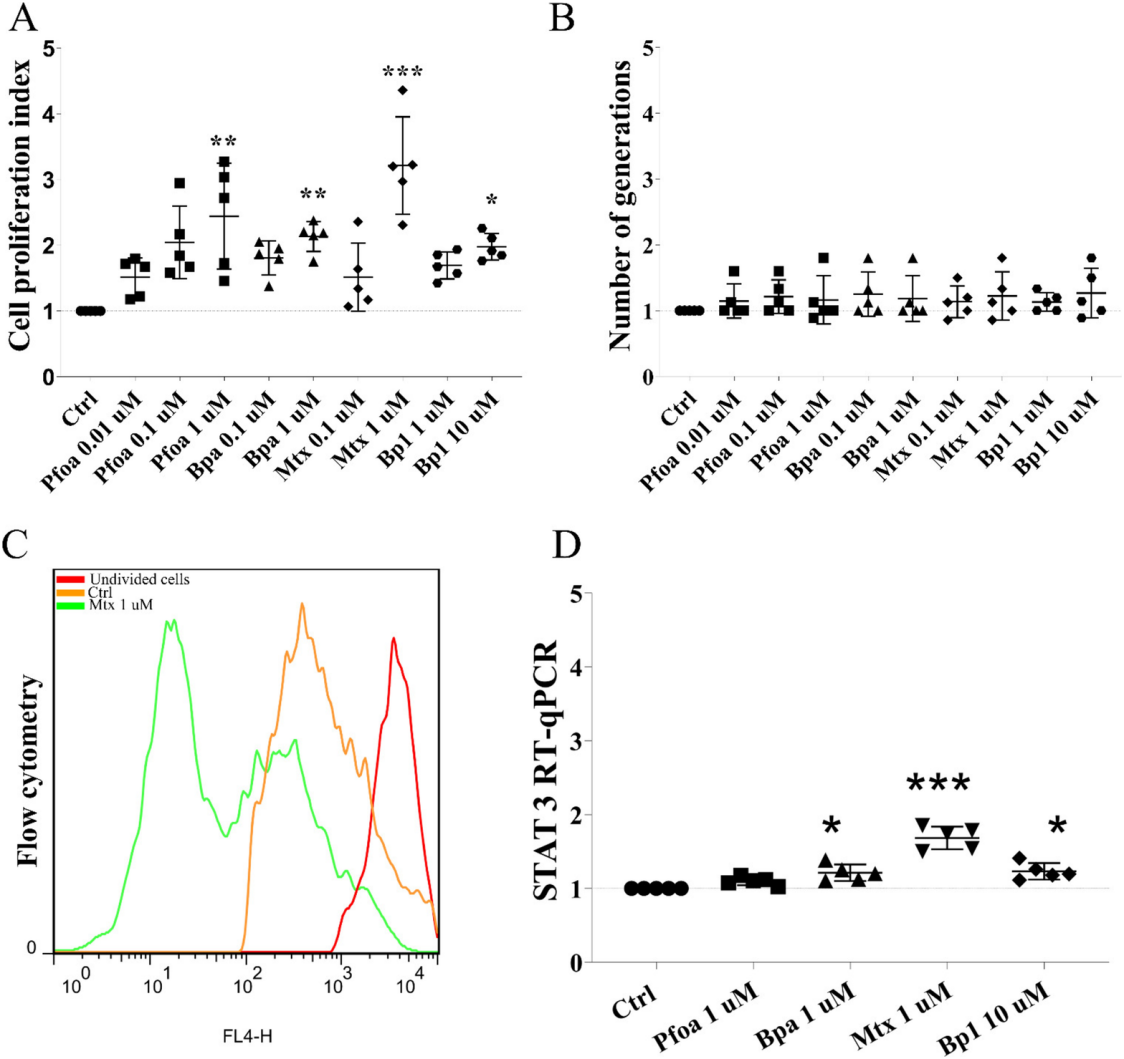
Cell proliferation and STAT3 expression in MCF7 tumor cells. Cell proliferation assay using CellTrace Far Red dye by flow cytometry and gene expression by RT‐qPCR. (A) Cell proliferation index. (B) Number of generations. (C) MCF7 cells labeled with CellTrace Far Red‐CFTR, red peak: cells that still did not divide (prior to any cell division), blue line: control (cells not exposed to the pollutants), orange line: cells exposed to the Mtx‐1 μM. (D) Expression of cell proliferation‐related gene, STAT3. 15d‐experiment. Horizontal line: mean, vertical line: SD (or 95% CI). One‐way ANOVA + Dunnett's test (D) or Kruskal–Wallis + Dunn's (A, B) post hoc test to compare treated cells with the respective control (dashed horizontal line). *p < 0.05, **p < 0.01, ***p < 0.001. *N* = 5 independent experiments (circles and polygons).

Expressive increases in the cell proliferation index occurred in the MCF7 tumor cells exposed to Pfoa‐0.1 μM (~100%) and 1 μM (~140%), Bpa‐1 μM (~110%), Mtx‐1 μM (~220%), and Bp1‐10 μM (~90%, Figure 3A–C) at the 15d experiment, although the number of generations that a cell has progressed since the fluorescent label was applied was not affected (Figure 3B). The expression of STAT3 (Signal transducer and activator of transcription 3), a key gene in the oncogenic pathway, also increased due to exposure to Bpa‐1 μM (~20%), Mtx‐1 μM (~60%), and Bp1‐10 μM (~20%, Figure 3D).

Taken together, the cell proliferation index and STAT3 gene expression corroborate the results of increases in neutral red uptake and cell attachment (crystal violet assay) and provide additional evidence of worsening of tumor cell phenotype.

### 3.5 Exposure to OP Increased Colony Formation and the Expression of Genes Associated With Tumor Progression

**FIGURE 4:**
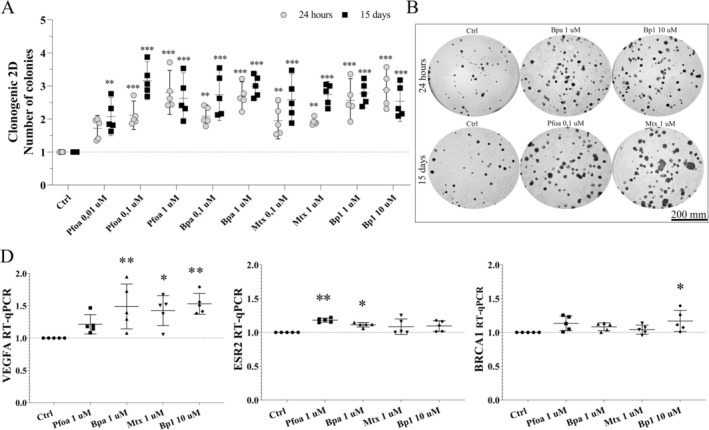
Cell colony formation and expression of genes associated with tumor progression in MCF7 tumor cells. (A) Cell colony formation (number of colonies) in solid substrate (2D clonogenic assay). Gray circles: 24 h‐experiment. Black squares: 15d‐experiment. (B) Images of colonies captured in a stereomicroscope. Scale bar = 200 mm. (C) Expression of genes related to tumor progression, VEGF, ESR2 and BRCA1 by RT‐qPCR for the 15d‐experiment. Horizontal line: mean, vertical line: SD (or 95% CI). One‐way ANOVA + Dunnett's test post hoc test to compare treated cells with the respective control (dashed horizontal line). *p < 0.05, **p < 0.01, ***p < 0.001. *N* = 5 independent experiments (circles and polygons).

The colony formation in solid substrate, which evaluates cell survival and proliferation at low density, increased in MCF7 cells exposed to the OP. For the 24 h‐experiment, increases were observed following exposure to Pfoa‐0.1 μM (~110%) and 1 μM (~180%), Bpa‐0.1 μM (~100%) and 1 μM (~160%), Mtx‐0.1 μM (~90%) and 1 μM (~90%), and Bp1‐1 μM (~150%) and 10 μM (~180%, Figure 4A,B). For the 15d‐experiment, increases in the number of colonies occurred after exposure to Pfoa‐0.01 μM (~100%), 0.1 μM (~210%) and 1 μM (~160%), Bpa‐0.1 μM (~170%) and 1 μM (~190%), Mtx‐0.1 μM (~150%) and 1 μM (~170%), and Bp1‐1 μM (~170%) and 10 μM (~150%, Figure 4A,B).

There was also an increase in the expression of different genes with great significance for cancer. Expression of VEGFA (Vascular endothelial growth factor A), the key modulator of angiogenesis, increased after exposure to Bpa‐1 μM (~40%), Mtx‐1 μM (~40%), and Bp1‐10 μM (~50%, Figure 4C). Likewise, the expression of ESR2 (Estrogen receptor 2), a gene usually associated with the response to treatment and breast cancer patients' survival, and of BRCA1 (Breast cancer type 1), a tumor suppressor gene associated with genomic stability and cell cycle, slightly increased (ESR2: Pfoa‐1 μM (~18%) and Bpa‐1 μM (~11%), BRCA1: Bp1‐10 μM (~16%), Figure 4C). Conversely, the OP did not affect the expression of the other genes: HSPA8 (Heat shock protein family A (Hsp70) member 8), SYNCRIP (Synaptotagmin‐binding, cytoplasmic RNA‐interacting protein), AKT1 (RAC (Rho family)‐alpha serine/threonine), AR (Androgen receptor), ESR1 (Estrogen receptor 1), MMP2 (Matrix metalloproteinase‐2), MMP9 (Matrix metalloproteinase‐9), SYMPK (Symplekin scaffold protein, Figure S2). Overall, the effects of OP on cell colony formation and VEGFA gene expression provide additional evidence of the negative outcomes of breast cancer cells' exposure to such chemicals.

### 3.6 Exposure to the OP Increased ABCG2 Gene Expression, but Not Drug Efflux Transporters Activity

**FIGURE 5:**
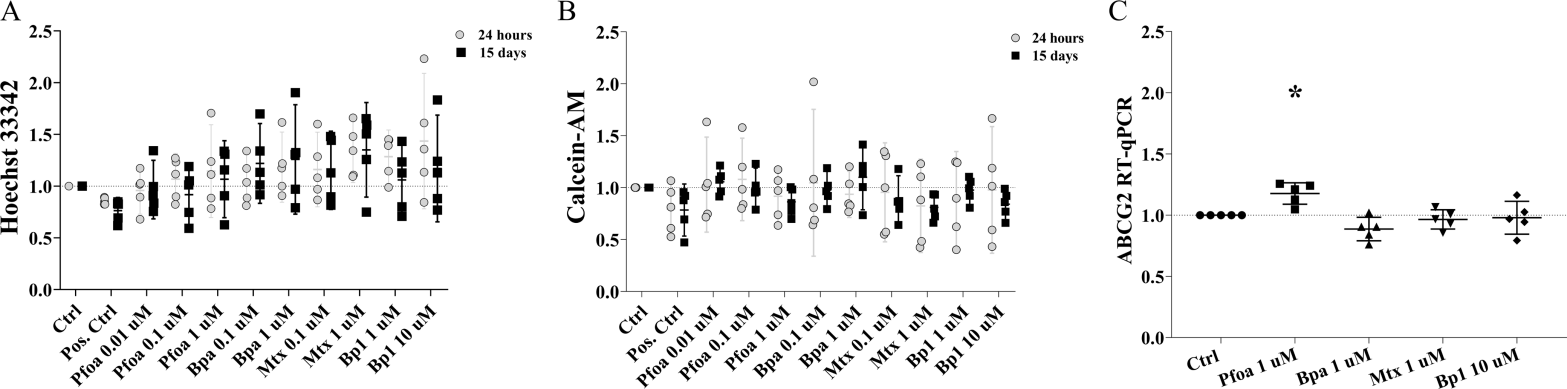
Drug efflux pumps activity and ABCG2 gene expression in MCF7 tumor cells. Drug efflux activity measured as fluorescent efflux from cells. (A) Hoechst 33342 (substrate for BCRPs), positive control: cells incubated with elacridar. (B) Calcein‐AM (substrate for MRPs), positive control: cells incubated with indomethacin. Gray circles: 24 h‐experiment. Black squares: 15d‐experiment. (C) ABCG2 gene expression by RT‐qPCR at 15d‐experiment. Horizontal line: mean, vertical line: SD (or 95% CI). One‐way ANOVA + Dunnett's test post hoc test to compare treated cells with the respective control (dashed horizontal line). *p < 0.05. *N* = 5 independent experiments (circles and polygons).

The efflux of the fluorescent solutes hoechst 33342, calcein‐AM (Figure 5A,B) and rhodamine B (Figure S1F) by ABC drug efflux pumps was not affected by the OP exposure at 24 h and 15 d experiments in MCF7 tumor cells. However, the expression of ABCG2 (ATP‐binding cassette super‐family G member 2), a gene that codes for a protein responsible for transporting a wide range of compounds, including chemotherapy drugs, out of the cells, increased after Pfoa‐1 μM (~17%) exposure at the 15 d experiment (Figure 5C).

Except for the increased ABCG2 gene expression by Pfoa exposure, it is a positive outcome that the other OP did not affect the activities of drug efflux pumps, as it limits the risk of developing the MDR (multidrug resistance) phenotype. Likewise, MCF7 cells are noninvasive cells and exposure to the OP did not reverse the nonmigratory feature of these cells (Figure S3).

### 3.7 Pan‐Cancer Analysis Using Transcriptomic Profiles to Evaluate Breast Cancer Cell Lines

**FIGURE 6:**
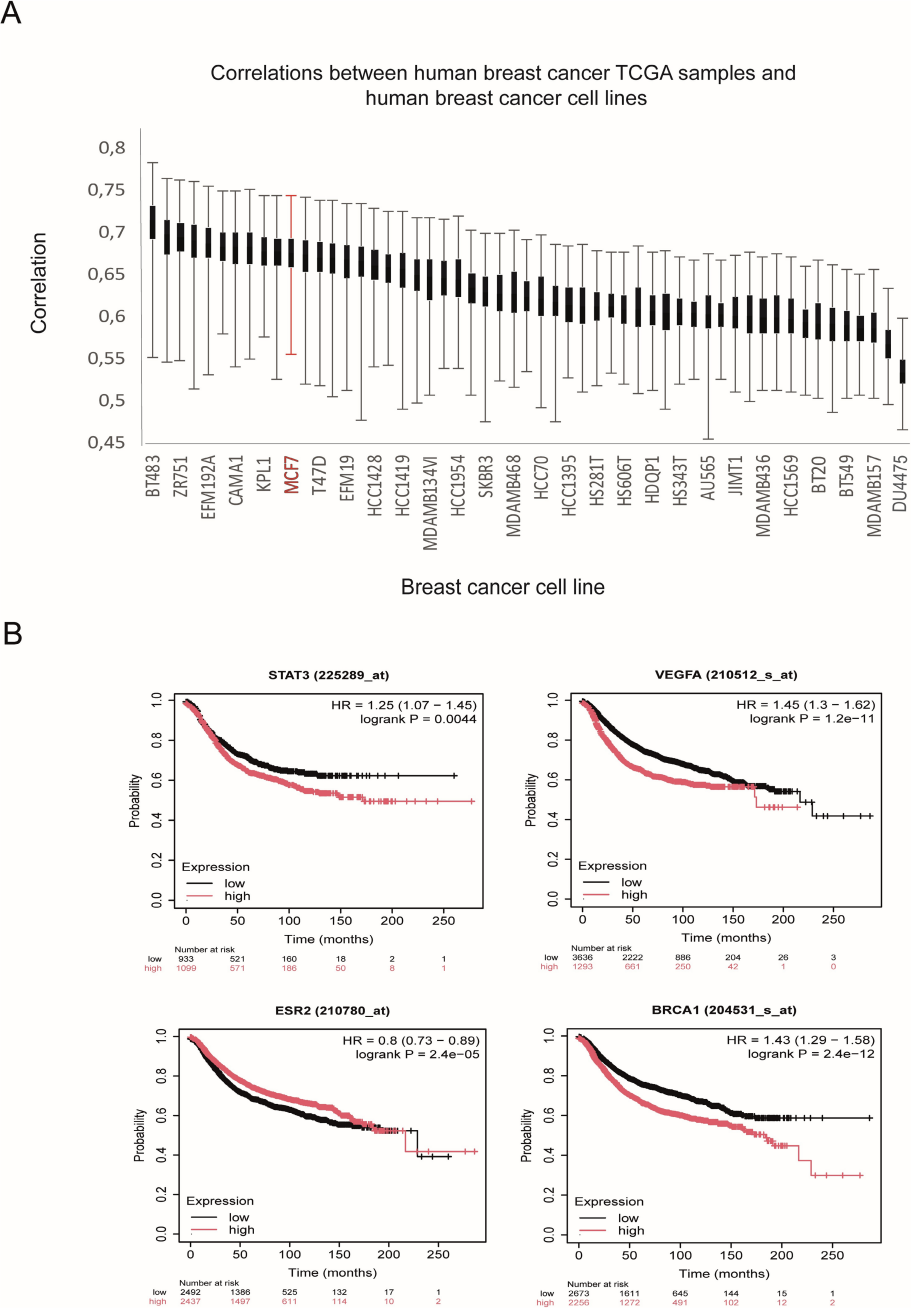
Spearman's correlations and survival analysis in breast cancer. (A) Boxplots of Spearman's correlations between breast cancer cell lines and human breast cancer tumor samples. The x‐axis represents different cell lines. A box plot is also shown, displaying the variation between the upper and lower limits for each cell line. The MCF7 cell line is highlighted in red among the 54 total cell lines. (B) Kaplan–Meier survival curves are provided for the BRCA1, ESR2, STAT3, and VEGF genes in breast cancer patients.

In order to investigate the common and unique characteristics of breast cancer cell lines for use as primary tumor models, pan‐cancer analysis was performed. After processing the data, Spearman correlation results between breast cancer cell lines and human breast cancer patients were generated. Gene expression and Kaplan–Meier survival analysis of the BRCA1, ESR2, STAT3, and VEGFA genes (genes whose expression increased after exposure to the OP) were performed using data from breast cancer patients. In the correlations between TCGA (The Cancer Genome Atlas) samples that refer to biological and clinical data collected from human breast cancer patients and 55 human breast cell lines, the MCF7 lineage is among the 11 main cell lines used as a primary tumor model for studies (Figure 6A). Also, the higher the gene expression of the BRCA1, STAT3, and VEGFA genes, the lower the survival rate of cancer patients (Figure 6B).

## 4 Discussion

The present study showed that low doses of the group of organic pollutants (OP)—perfluorooctanoic acid (PFOA), bisphenol A (BPA), methoxychlor (MTX), and benzophenone‐1 (BP1)—can promote a more aggressive phenotype in human breast cancer MCF7 cells. In this regard, molecular docking proved very useful in helping to select the compounds to be tested in the in vitro experiments, as observed by their effects on tumor cells. Overall, the OP altered cellular characteristics associated with increased tumor progression in MCF7 cells, such as cell proliferation, colony‐forming ability, and the expression of key cancer‐related genes. These findings reinforce the need for a new strategy to investigate the role of environmental contaminants in disease development and chemotherapeutic response.

At the beginning of the study, some signs of modulation of MCF7 (tumor) and MCF10A (nontumor) cells by OP exposure were already observed, such as increased neutral red (NR) uptake, MTT metabolism by cellular dehydrogenases, and cell adhesion, particularly after long‐term exposure (15 days) in MCF7 cells. These findings may be associated with an increased number of cells due to enhanced proliferation, decreased cell death, or both, and indicate that the tested concentrations of OP, reported in human blood plasma (Michałowicz 2014; Cabrera‐Rodríguez et al. 2018; Arbuckle et al. 2013; Wang et al. 2016), were not cytotoxic in vitro. In particular, the increased neutral red uptake may also indicate autophagy, an important process that provides nutrients to support tumor cell survival under stress conditions (Zhang et al. 2021; Davidson and Vander Heiden 2017). Overall, the effects on MCF10A cells were smaller than those on tumor cells and were observed during short‐term exposure (24 h), indicating that cancer cells, which already display aberrant behavior, are more prone to respond to exposure to low OP concentrations. This is a concern for risk assessment, as most studies on pollutants tend to focus on cytotoxic effects in models targeting normal cells rather than aberrant ones.

Considering the indication of an increased number of cells by the colorimetric assays (NR, MTT, and CV), a specific cell proliferation assay (CellTrace) was performed for the 15‐day experiment. OP increased the cell proliferation index in both cell lines (MCF7 and MCF10A) but not the number of cell generations. These two pieces of data have different meanings: a high proliferation index indicates that a large proportion of cells are dividing, suggesting a high cell growth rate, whereas the number of generations indicates how many divisions occurred from the ancestral cells (i.e., those present at the start of the assay) to the current cell population. Therefore, OP exposure may increase the number of cells entering and completing the cell cycle, which could have important consequences in vivo, leading to an increased tumor growth rate. Again, the effects were more pronounced in tumor cells (MCF7) than in their normal counterpart (MCF10A), indicating that cells with “defective” cell cycle control (e.g., tumor cells) may respond more readily to a pro‐mitogenic effect of OP. Further confirmation of cell proliferation came from the colony formation (clonogenic or plating efficiency) assay.

Colony formation mainly depends on the ability of cells to survive at low density and proliferate (Franken et al. 2006), which can be challenging for many cells that die at low density. OP exposure had two major effects in this regard: it increased the number of surviving cells and the size of colonies due to enhanced cell proliferation. Some studies have already reported that OP exposure increases proliferative capacity (Lee et al. 2012; Park et al. 2013; In et al. 2015; Deng et al. 2021) and colony formation (Qu et al. 2023) in tumor cells, but usually at higher concentrations than those used in our study. This indicates that the risk of pollutant‐induced tumor proliferation should be a major concern in cancer.

Cell proliferation in hormone‐responsive cells, such as MCF7, can be regulated by hormones such as estrogen and by ESR1 and ESR2 receptors (Xiang et al. 2024). This raises concern about exposure to endocrine disruptors, compounds that can mimic or inhibit the actions of natural hormones. Some compounds, such as BPA, PFOA, organochlorines, and benzophenones, display strong estrogenic activity and affect several physiological processes in estrogen‐responsive organs (Lecomte et al. 2017).

In the present study, exposure to PFOA and BPA increased ESR2 expression in MCF7 cells. ESR2 activity increases endothelial cell proliferation, neovascularization, and angiogenesis, which may consequently enhance tumor growth and metastatic potential. In particular, tumor cell survival and proliferation depend on nutrients supplied by the blood, making angiogenesis a key process required for continued tumor growth. In this context, regulation of the pro‐angiogenic factor VEGF may represent one of the main molecular pathways responsible for the angiogenic effect of ESR2 during breast cancer development and progression (Rajoria et al. 2011; Arnal et al. 2017). Increased ESR2 suggests potential activation of signaling pathways such as AKT/mTOR and JAK/STAT, previously identified as mediators of cell proliferation, invasion, and therapeutic resistance (Mangani et al. 2024). Thus, ERβ gain in response to contaminants may favor a more invasive phenotype and reduced responsiveness to endocrine therapy. Moreover, exposure to BPA, MTX, and BP1 increased VEGFA gene expression in MCF7 cells, which correlates with an unfavorable cancer context, since this gene encodes a protein that induces endothelial cell proliferation, inhibits apoptosis, and promotes cell migration (Rajoria et al. 2011).

The VEGFA gene plays an important role in tumor aggressiveness, being linked to increased cell proliferation. Its overexpression has been associated with higher expression of the Ki67 marker, higher Nottingham histological grades, and enrichment of Hallmark gene sets linked to proliferation, such as MYC v1/v2 targets, E2F targets, G2M checkpoint, mitotic spindle, and mTORC1 signaling (Sharma et al. 2025). Beyond its classical role in angiogenesis, VEGFA promotes cell proliferation and migration, particularly in endothelial cells, which express high levels of VEGFRs (Al Kawas et al. 2022). Evidence suggests that VEGFA increases the population of tumor‐initiating stem cells in murine and human models and induces epithelial‐mesenchymal transition (EMT) and metastasis (Kim et al. 2017).

Expression of two other key cancer genes, STAT3 and BRCA1, also increased after OP exposure (STAT3: BPA, MTX, and BP1; BRCA1: BP1). STAT3 plays an important role in cell survival, proliferation, migration, and differentiation (Wang et al. 2023). Furthermore, STAT3‐induced activation of vascular endothelial growth factor (VEGF) and proliferation‐associated genes is linked to tumor development and progression (Rodriguez‐Barrueco et al. 2015; Lin et al. 2020; Heichler et al. 2020). The STAT3 signaling pathway plays a central role in regulating cellular processes in breast cancer. Bisphenol A (BPA) can induce STAT3 expression in MCF7 cells exposed to 1 μM and changes in the phosphorylation state (Zhang et al. 2012). STAT3 is activated via growth factor receptor signaling, recruiting Src and JAKs to phosphorylate tyrosine residues, or via receptor tyrosine kinases (EGFR, VEGFR, PDGFR, CSF‐1R) and nonreceptor kinases such as Src and ABL. After phosphorylation, STAT3 dimerizes, translocates to the nucleus, and regulates genes related to proliferation, survival, and metastasis, including BCL2, BIRC5, c‐Myc, CCND1, and VEGF. In breast cancer, constitutive STAT3 activation promotes proliferation, drug resistance, angiogenesis, and apoptosis evasion (Wang et al. 2023; Rodrigues et al. 2024; Jiang et al. 2024). STAT3 phosphorylation is also linked to apoptosis inhibition, while its inhibition can induce P53 expression and trigger senescence and apoptosis. In other tumors, such as melanoma, STAT3 supports angiogenesis and metastasis via bFGF and VEGF (Wang et al. 2023). STAT3 activation can additionally be modulated by reactive oxygen species, depending on the redox state of breast cancer cells (Rodrigues et al. 2024), and it directly regulates PD‐L1 expression, reinforcing its role in tumor immunosuppression (Temple and Walker 2025).

BRCA1 plays relevant roles in genomic stability and cellular homeostasis, influencing several processes such as cell differentiation (Zhong et al. 2023). Higher BRCA1 expression may reflect an adaptive response to genome stress induced by pollutants, enhancing DNA repair capacity and allowing cells with genetic lesions to continue proliferating. This increase was associated with the enrichment of gene sets linked to cell proliferation, including E2F targets, MYC, G2M checkpoint, and mitotic spindle (Chida et al. 2024), suggesting that BRCA1 may act as a facilitator of tumor growth in an already transformed context. These findings support the hypothesis that OP may modulate the MCF7 cell phenotype toward a more aggressive state, increasing the expression of ESR2, VEGFA, and STAT3 genes. In this regard, pan‐cancer analysis revealed that increased expression of ESR2, VEGFA, and STAT3, but not BRCA1, contributes to decreased survival rates in breast cancer patients.

Surgery followed by chemotherapy is one of the main strategies used for breast cancer patients. However, the development of multidrug resistance (MDR) is a challenge that can render chemotherapy ineffective. This phenomenon is characterized by tumor cell resistance to a wide range of drugs (Assaraf et al. 2019; Li et al. 2016), usually due to enhanced activity of drug efflux pumps. Among the ABC pumps involved in MDR, the breast cancer resistance protein (BCRP), encoded by the ABCG2 gene, stands out (Mao and Unadkat 2015). In the present study, OP exposure did not affect the efflux activity of PgP, MRPs, and BCRP in MCF7 cells, although ABCG2 expression was slightly increased (PFOA). Considering the efflux activity results, possible post‐transcriptional regulation, and the modest increase in ABCG2 expression, there is no evidence that OP may affect MCF7 cell resistance to chemotherapeutic drugs through MDR induction, which is positive in the cancer context. However, some of our recent studies have reported that exposure to other pollutants modulated drug efflux transporter expression in B16‐F1 murine melanoma cells, such as polybrominated diphenyl ethers (BDE‐47 and BDE‐209), tetrabromodibenzo‐p‐dioxin (TCDD), and some pesticide mixtures (De Souza Salgado et al. 2018; De Marchi et al. 2021; Steil et al. 2022; De Almeida Roque et al. 2023), so the possibility of MDR induction cannot be ruled out for other chemicals and cell models.

Finally, OP exposure may sensitize nontumor MCF10A cells by partially decreasing efflux transporter activity. In healthy tissues, these transporters form important physiological barriers, such as the blood–brain barrier, protect cells against toxic xenobiotics, and transport some endogenous metabolites, including inflammatory mediators (Nedeljković et al. 2021). However, this protective effect was temporarily impaired by OP, as a reduction in calcein‐AM efflux (due to decreased MRP activity) occurred after short‐term exposure (24 h, PFOA, BPA, MTX, and BP1), although it was generally compensated for after long‐term exposure (15 days). This decrease in activity could be a problem if it occurs in vivo, as more significant side effects (in normal cells) may arise during chemotherapy in cancer patients.

## 5 Conclusion

In the present study, we have shown that Pfoa, Bpa, Mtx, and Bp1 increase the malignant phenotype of human breast cancer MCF7 cells by increasing the expression of some key genes ESR2, VEGFA, STAT3, and ABCG2, and their proliferative and low‐density survival capacity in vitro. Given the widespread domestic and industrial use of these chemicals and respecting the appropriate proportions and extrapolations from in vitro models, the effects reported in the present and previous studies should raise concerns about the risk of exposure in cancer patients.

## Conflicts of Interest

The authors declare no conflicts of interest.

## Supporting information


**Data S1:** Supplementary Material.

**Figure S1:** Cell viability, proliferation and drug‐efflux transporters activity in MCF10A (A‐E) and MCF7 (F) cells. A: Rhodamine B (substrate for PgP/MRPs), positive control: cells incubated with verapamil (MCF10A cells). B: Hoechst 33342 (substrate for BCRPs), positive control: cells incubated with elacridar. C: Cell attachment/crystal violet (CV) assay. D: MTT metabolism (MTT) assay. E: number of generations. F: Rhodamine B (substrate for PgP/MRPs), positive control: cells incubated with verapamil (MCF7 cells). Gray circles: 24 h‐experiment. Black squares: 15d‐experiment. Horizontal line: mean, vertical line: SD (or 95% CI). Kruskal‐Wallis + Dunn's (D) post hoc test to compare the cells exposed to the OP with the respective control (dashed horizontal line). **p < 0.01, ***p < 0.001. *N* = 5 independent experiments (circles and polygons).

**Figure S2:** Expression of genes related to tumor progression in MCF7 tumor cells. A: HSPA8. B: RPS6. C: SYNCRIP. D: AKT1. E: AR. F: ESR1. G: 𝛃‐TUBULIN. H: MMP2. I: MMP9. RT‐qPCR for the 15d‐experiment. Horizontal line: mean, vertical line: SD (or 95% CI). One‐way ANOVA. Control: dashed horizontal line. *N* = 5 independent experiments (circles and polygons).

**Figure S3:** Cell migration by scratch assay. A‐B: MCF7 tumor cells after the 24 h‐experiment. C‐D: MCF7 tumor cells after the 15d‐experiment. E‐F: MCF10A nontumor cells after 24 h‐experiment. G‐H: MCF10A nontumor cells after 15d‐experiment. Horizontal line: mean, vertical line: SD (or 95% CI). One‐way ANOVA. Control: dashed horizontal line. *N* = 5 independent experiments (circles and polygons).

## Data Availability

The data that support the findings of this study are available from the corresponding author upon reasonable request.
