# Supplementary material for: Impact of organic pollutants on phenotype and gene expression in human breast cancer cells
Source: J Appl Toxicol. 2025 Oct 21;46(5):1487–505. doi: 10.1002/jat.4961 (PMC13040441; doi:10.1002/jat.4961)
Supplement: Supplementary file 1 — Data S1: Supplementary Material. [file JAT-46-1487-s002.docx]

Supplementary figure 1. Cell viability, proliferation and drug-efflux transporters activity in MCF10A (A-E) and MCF7 (F) cells. A: Rhodamine B (substrate for PgP/MRPs), positive control: cells incubated with verapamil (MCF10A cells). B: Hoechst 33342 (substrate for BCRPs), positive control: cells incubated with elacridar. C: Cell attachment/crystal violet (CV) assay. D: MTT metabolism (MTT) assay. E: number of generations. F: Rhodamine B (substrate for PgP/MRPs), positive control: cells incubated with verapamil (MCF7 cells). Gray circles: 24h-experiment. Black squares: 15d-experiment. Horizontal line: mean, vertical line: SD (or 95% CI). Kruskal-Wallis + Dunn's (D) post hoc test to compare the cells exposed to the OP with the respective control (dashed horizontal line). **p<0.01, ***p<0.001. N=5 independent experiments (circles and polygons).

Supplementary figure 2. Expression of genes related to tumor progression in MCF7 tumor cells. A: HSPA8. B: RPS6. C: SYNCRIP. D: AKT1. E: AR. F: ESR1. G: 𝛃- TUBULIN. H: MMP2. I: MMP9. RT-qPCR for the 15d-experiment. Horizontal line: mean, vertical line: SD (or 95% CI). One-way ANOVA. Control: dashed horizontal line. N=5 independent experiments (circles and polygons).

Supplementary figure 3. Cell migration by scratch assay. A-B: MCF7 tumor cells after the 24h-experiment. C-D: MCF7 tumor cells after the 15d-experiment. E-F: MCF10A non-tumor cells after 24h-experiment. G-H: MCF10A non-tumor cells after 15d-experiment. Horizontal line: mean, vertical line: SD (or 95% CI). One-way ANOVA. Control: dashed horizontal line. N=5 independent experiments (circles and polygons).
