# Supplementary figures and images for: Impact of organic pollutants on phenotype and gene expression in human breast cancer cells
Source: J Appl Toxicol. 2025 Oct 21;46(5):1487–505. doi: 10.1002/jat.4961 (PMC13040441; doi:10.1002/jat.4961)

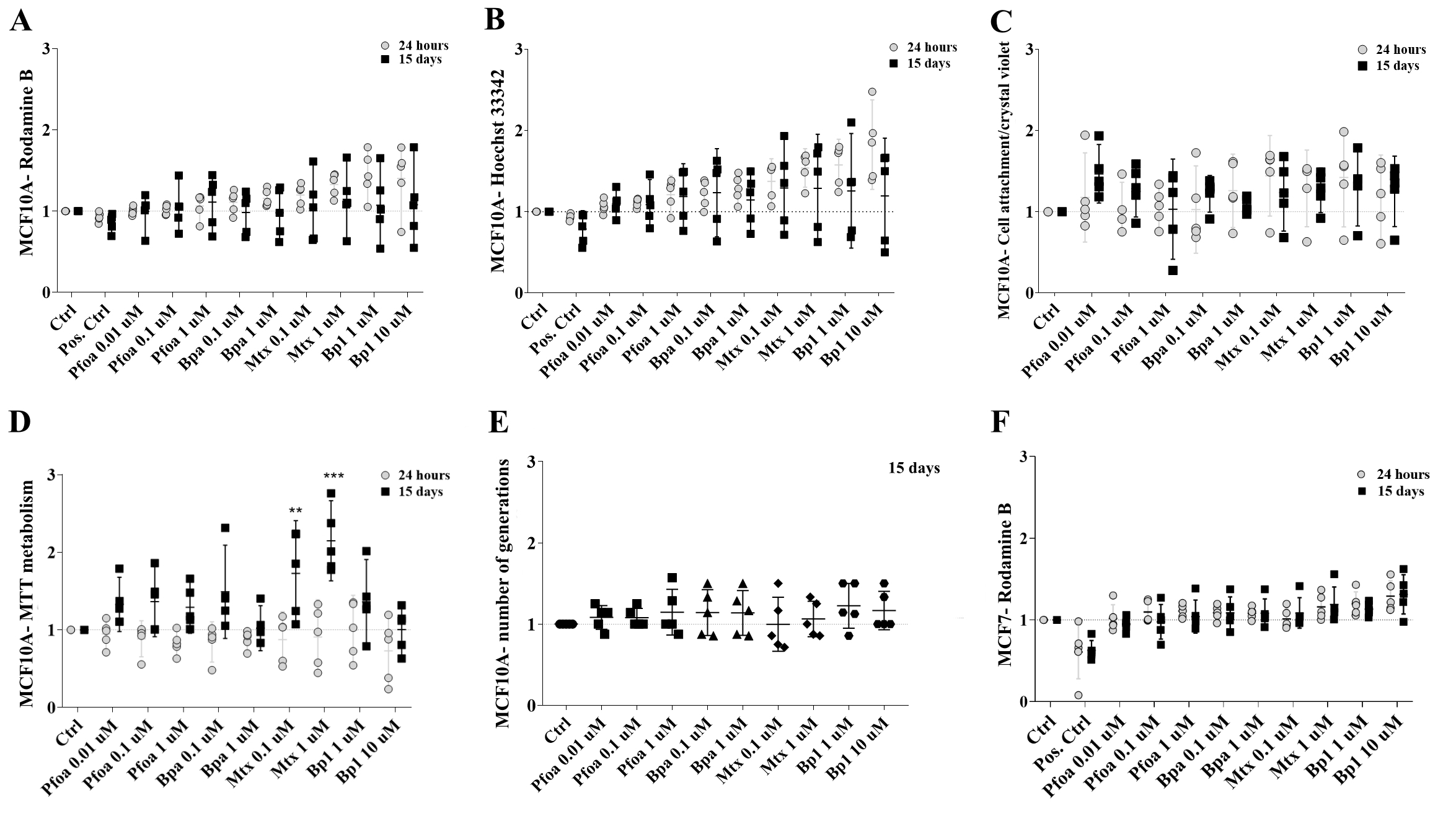

Supplement: Supplementary file 2 — Figure S1: Cell viability, proliferation and drug‐efflux transporters activity in MCF10A (A‐E) and MCF7 (F) cells. A: Rhodamine B (substrate for PgP/MRPs), positive control: cells incubated with verapamil (MCF10A cells). B: Hoechst 33342 (substrate for BCRPs), positive control: cells incubated with elacridar. C: Cell attachment/crystal violet (CV) assay. D: MTT metabolism (MTT) assay. E: number of generations. F: Rhodamine B (substrate for PgP/MRPs), positive control: cells incubated with verapamil (MCF7 cells). Gray circles: 24 h‐experiment. Black squares: 15d‐experiment. Horizontal line: mean, vertical line: SD (or 95% CI). Kruskal‐Wallis + Dunn's (D) post hoc test to compare the cells exposed to the OP with the respective control (dashed horizontal line). **p < 0.01, ***p < 0.001. N = 5 independent experiments (circles and polygons). [file JAT-46-1487-s003.jpg]
